# Effects of Paracetamol (Acetaminophen) Ingestion on Endurance Performance: A Systematic Review and Meta-Analysis

**DOI:** 10.3390/sports9090126

**Published:** 2021-09-06

**Authors:** Jozo Grgic, Pavle Mikulic

**Affiliations:** 1 Institute for Health and Sport, Victoria University, Melbourne 3011, Australia; 2 Faculty of Kinesiology, University of Zagreb, 10000 Zagreb, Croatia; pavle.mikulic@kif.unizg.hr

**Keywords:** ergogenic effects, data synthesis, aerobic endurance

## Abstract

Several studies explored the effects of paracetamol (acetaminophen) ingestion on endurance performance, but their findings are conflicting. Therefore, this review aimed to conduct a meta-analysis examining the effects of paracetamol ingestion on endurance performance. Five databases were searched to find relevant studies. The PEDro checklist was used to assess the methodological quality of the included studies. Data reported in the included studies were pooled in a random-effects meta-analysis. A total of ten studies with good or excellent methodological quality were included in the meta-analysis (pooled *n* = 141). All included studies had a randomized, double-blind, crossover design. In the main meta-analysis, there was no significant difference between the effects of placebo and paracetamol on endurance performance (Cohen’s *d* = 0.09; 95% confidence interval (CI): −0.04, 0.22; *p* = 0.172). However, an ergogenic effect was found when we considered only the studies that provided paracetamol 45 to 60 min before exercise (Cohen’s *d =* 0.14; 95% CI: 0.07, 0.21; *p* < 0.001). In a subgroup analysis that focused on time-to-exhaustion tests, there was a significant ergogenic effect of paracetamol ingestion (Cohen’s *d* = 0.19; 95% CI: 0.06, 0.33; *p* = 0.006). There was no significant difference between placebo and paracetamol in a subgroup analysis that focused on time trial tests (Cohen’s *d* = 0.05; 95% CI: −0.12, 0.21; *p* = 0.561). In conclusion, paracetamol ingestion appears to enhance performance (a) in time-to-exhaustion endurance tests and (b) when consumed 45 to 60 min before exercise.

## 1. Introduction

Paracetamol (acetaminophen) is one of the most popular drugs for pain relief and fever reduction [1,2]. Paracetamol primarily acts by inhibiting prostaglandin synthesis, which reduces transduction of the sensory nerves, resulting in decreased nociceptive impulse transmission [3]. Besides the general population, paracetamol is also consumed by athletes [4,5]. For example, a study conducted among 141 young sub-elite athletes reported that paracetamol was detected in urine samples of 9.5% of all participants [4]. Similar to these findings, in a cohort of 98 young regional-to-national-level athletes, Garcin et al. [5] reported that paracetamol was detected during urinary screening for doping substances in 9.2% of the participants. The most commonly reported reason for consuming paracetamol among athletes is to decrease pain from previous athletic exertion [4,5,6]. Currently, paracetamol use is not prohibited by the World Anti-Doping Agency. 

While paracetamol is used in sport and exercise to reduce pain from a previous exercise bout, less is known about its acute ergogenic effects [7,8,9,10,11,12,13,14,15,16,17,18,19,20]. Acute muscle pain occurs during different forms of endurance exercise (e.g., middle distance running and cycling) [21]. It has been proposed that exercise-induced pain tolerance is an important factor in endurance performance [22,23]. One recent study [22] found that exercise-induced pain tolerance is significantly correlated with cycling performance (*r* = 0.83). Accordingly, researchers have devoted their attention to strategies that ameliorate the perceptions of pain during exercise to improve endurance performance [23]. Theoretically, lowering pain during endurance tasks by consuming paracetamol could improve exercise performance. One study provided a placebo or paracetamol dose of 1500 mg 60 min before a 16.1 km cycling time trial to a sample of trained male cyclists [10]. This study reported that acute ingestion of paracetamol reduced the time needed to complete the cycling event by 45 s [10]. However, more recent studies [9,15,16] used similar protocols and exercise tests but did not observe an ergogenic effect of paracetamol on endurance performance. In one example, Jessen et al. [9] reported that power output during 6 min of cycling was similar following the ingestion of placebo (312 ± 41 W) and 1500 mg of paracetamol (313 ± 45 W).

Due to the conflicting reports, it is currently difficult to provide a conclusive recommendation regarding the ergogenic effects of paracetamol on endurance performance. However, expanding our knowledge on this topic would be practically relevant because some reports indicate paracetamol consumption among athletes [4,5]. Therefore, we aimed to conduct a meta-analysis examining the effects of paracetamol ingestion on endurance performance.

## 2. Materials and Methods

### 2.1. Search Strategy

In the primary part of the search process, we searched through five databases: Networked Digital Library of Theses and Dissertations, PubMed/MEDLINE, SPORTDiscus, Scopus, and Web of Science. We used the following search syntax in all databases: (paracetamol OR acetaminophen) AND (“time trial” OR “time to exhaustion” OR running OR “exercise performance” OR “mean power” OR endurance OR “volitional fatigue”). In the secondary search, we examined the references list of all included studies and conducted forward citation tracking through Google Scholar. The search for studies was performed on 5 August 2021. The search was performed independently by the two authors of the review. 

### 2.2. Inclusion Criteria

We included studies that satisfied the following criteria: (1) examined the effects of paracetamol ingestion on endurance performance; (2) used a double-blind, crossover, and placebo-controlled study design; (3) included humans as study participants.

### 2.3. Data Extraction

From all included studies, we extracted the following data: (1) lead author name and year of publication; (2) participants’ characteristics; (3) protocol of paracetamol ingestion (e.g., dose and the timing of ingestion); (4) endurance test; (5) mean ± standard deviation from the endurance test following placebo and paracetamol ingestion. One study [16] presented mean ± standard deviation data in a figure. For this study, we used the Web Plot Digitizer software (https://apps.automeris.io/wpd/ (accessed on 6 August 2021)) to extract the necessary data.

### 2.4. Methodological Quality 

We used the PEDro checklist to assess the methodological quality of the included studies [24]. This checklist utilizes 11 items evaluating various methodological aspects, including randomization, inclusion criteria, blinding, allocation concealment, attrition, and data reporting. Every item on the PEDro checklist is scored as “1” (criterion is satisfied) or “0” (criterion is not satisfied). However, the first item (“eligibility criteria were specified”) does not contribute to the total score. Therefore, the maximum possible number of points that can be scored on the PEDro checklist is 10. Based on this score, studies are classified as excellent, good, fair, and poor methodological quality if they scored 9–10 points, 6–8 points, 4–5 points, and ≤3 points, respectively [25,26].

### 2.5. Statistical Analysis

The meta-analysis was performed using standardized mean differences (Cohen’s *d*). Cohen’s *d* effect sizes were calculated using endurance performance mean ± standard deviation data from the placebo and paracetamol trials (i.e., the difference in means divided by the pooled standard deviation), total sample size, and correlation between the trials. Given that none of the studies reported correlation, we have estimated these values as suggested in the Cochrane Handbook [27]. Sensitivity analyses were performed by examining the pooled results after excluding one study at a time. As we detected high heterogeneity in the main meta-analysis (*I*^2^ = 79%), we have performed additional analyses to explore the reasons for this heterogeneity. In one analysis, we only considered the data from studies that provided paracetamol 45 to 60 min before exercise. Additionally, subgroup meta-analyses explored the effects of paracetamol ingestion on performance in time-to-exhaustion tests vs. time trials. Effect sizes were interpreted as trivial (<0.20), small (0.20–0.49), medium (0.50–0.79), and large (≥0.80) [28]. All meta-analyses were performed using the random-effects model. Heterogeneity was explored using the *I*^2^ statistic, which was interpreted as low (<50%), moderate (50–75%), and high heterogeneity (>75%). The statistical significance threshold was set at *p* < 0.05. All analyses were performed using the Comprehensive Meta-Analysis software, version 2 (Biostat Inc., Englewood, NJ, USA). 

## 3. Results

### 3.1. Search Results 

**Figure 1:**
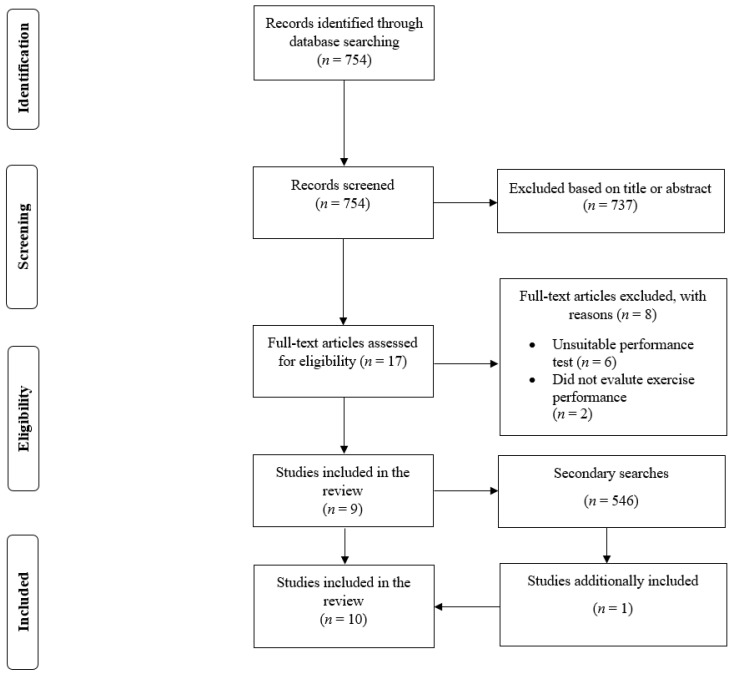
Flow diagram of the search process.

In the primary search, there were 754 results (Figure 1). From this number, we read 17 full-text papers and included nine studies [7,9,10,11,12,13,14,15,16]. In the secondary search, there were another 546 results. The secondary search resulted in the inclusion of one additional study [8]. Therefore, a total of ten studies were included in the review [7,8,9,10,11,12,13,14,15,16].

### 3.2. Summary of Studies

The included studies were published between 2010 and 2021. Sample sizes varied from 7 to 29 participants. The pooled number of participants across the ten included studies was 141. All of the studies included males as participants that were either recreationally active or competitive athletes. Four studies used a paracetamol dose of 1500 mg [9,10,14,16], two studies [7,8] used 500 mg, and one study [12] used 1000 mg. Three studies [11,13,15] provided paracetamol in relative doses, using 20 mg per kg of body mass or per kg of lean body mass. Paracetamol was ingested 60 min before exercise in six studies [8,9,10,11,12,15], 45 min before exercise in two studies [13,14], and 120 min before exercise in two studies [7,16]. The endurance tests used in the included studies are summarized in Table 1. Four studies used running- or cycling-to-exhaustion tests, whereas others used running or cycling time trials.

### 3.3. Methodological Quality

The included studies scored from 8 to 9 points on the PEDro checklist. Nine studies were classified as being of excellent methodological quality, while one study [9] was classified as being of good methodological quality.

### 3.4. Meta-Analysis Results

**Figure 2:**
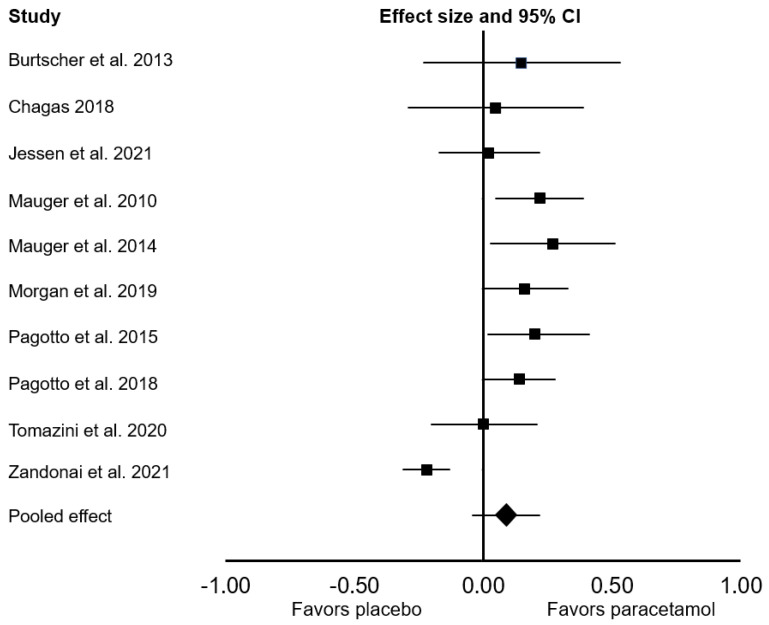
Forest plot presenting the results of the random-effects meta-analysis comparing the effects of placebo vs. paracetamol on endurance performance. Data are reported as Cohen’s *d* (effect size) and 95% confidence interval (CI). The diamond at the bottom presents the overall effect. The plotted squares denote effect sizes, and the whiskers denote their 95% CIs.

**Figure 3:**
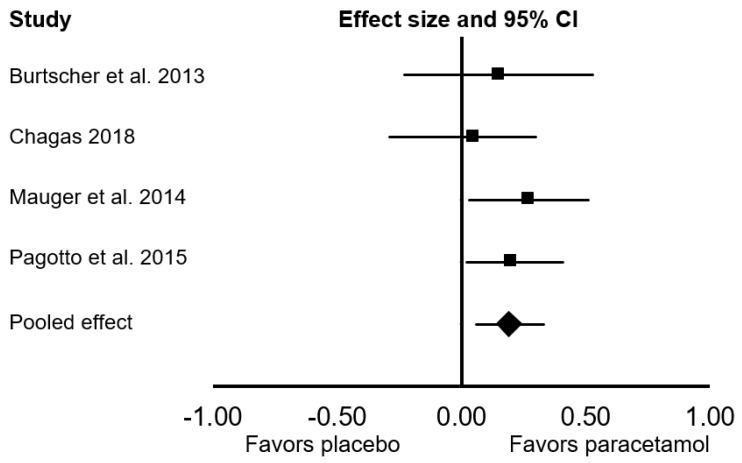
Forest plot presenting the results of the random-effects meta-analysis comparing the effects of placebo vs. paracetamol on endurance performance in time-to-exhaustion tests. Data are reported as Cohen’s *d* (effect size) and 95% confidence interval (CI). The diamond at the bottom presents the overall effect. The plotted squares denote effect sizes, and the whiskers denote their 95% CIs.

**Figure 4:**
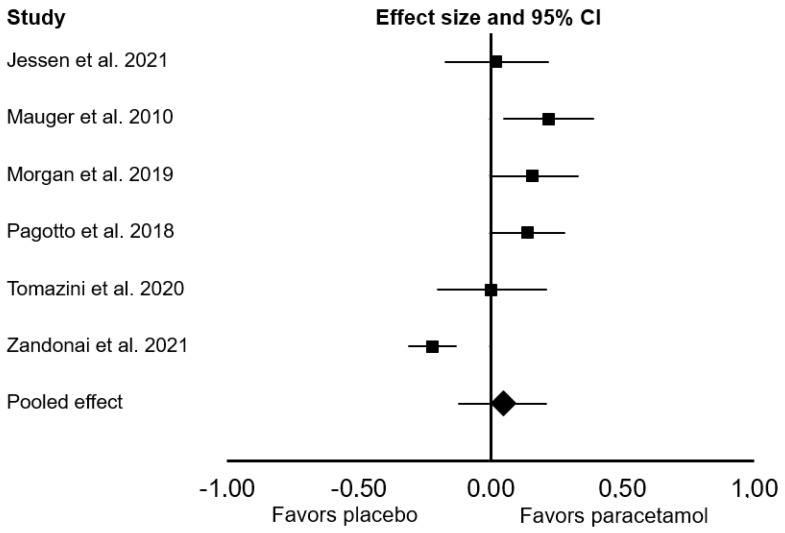
Forest plot presenting the results of the random-effects meta-analysis comparing the effects of placebo vs. paracetamol on endurance performance in time trial tests. Data are reported as Cohen’s *d* (effect size) and 95% confidence interval (CI). The diamond at the bottom presents the overall effect. The plotted squares denote effect sizes, and the whiskers denote their 95% CIs.

All ten studies were included in the main meta-analysis. There was no significant difference between the effect of placebo and paracetamol on endurance performance (Cohen’s *d* = 0.09; 95% confidence interval (CI: −0.04, 0.22; *p* = 0.172; *I*^2^ = 79%; Figure 2). In the sensitivity analyses, the exclusion of one study [16] had a meaningful effect on the pooled results, changing the pooled Cohen’s *d* to 0.14 (95% CI: 0.07, 0.20; *p* < 0.001; *I*^2^ = 0%). When we considered only studies that provided paracetamol 45 to 60 min before exercise, there was a significant ergogenic effect on endurance performance (Cohen’s *d*: 0.14; 95% CI: 0.07, 0.21; *p* < 0.001; *I*^2^ = 0%). In a subgroup analysis that focused only on endurance performance in time-to-exhaustion tests, there was a significant ergogenic effect of paracetamol ingestion (Cohen’s *d*: 0.19; 95% CI: 0.06, 0.33; *p* = 0.006; *I*^2^ = 0%; Figure 3). In a subgroup analysis that focused only on endurance performance in time trial tests, there was no significant difference between placebo and paracetamol (Cohen’s *d*: 0.05; 95% CI: −0.12, 0.21; *p* = 0.561; *I*^2^ = 85%; Figure 4). 

## 4. Discussion

When we pooled the data from all available studies, this meta-analysis did not find a significant difference between the effects of placebo and paracetamol on endurance performance. However, it appears that the effects of paracetamol are moderated by the timing of ingestion and the performance test. Specifically, paracetamol was ergogenic for endurance performance when ingested from 45 to 60 min before exercise. Furthermore, we also found that paracetamol enhances performance in cycling or running time-to-exhaustion tests but not in time trials. Still, it should be mentioned that these effects were in the range of a trivial to small magnitude. 

Studies have indicated that the plasma half-life of paracetamol is around 1.5 to 2.5 h [29,30]. However, two studies [7,16] included in the presented review provided paracetamol 120 min before exercise. These studies did not record an ergogenic effect and—due to its half-life—this is not likely to be the optimal timing of paracetamol ingestion. Indeed, when we considered data from studies that used 45 to 60 min before exercise as the timing of ingestion, there was a significant ergogenic effect of paracetamol. Thus, based on the data presented herein, it appears that the timing of ingestion may modulate the ergogenic effects of paracetamol ingestion on endurance performance. Specifically, it appears that the optimal timing of paracetamol ingestion is likely to be around 30 to 60 min before exercise. However, all of the included studies used only one timing of paracetamol ingestion, which highlights the need for future studies to directly explore the effects of paracetamol timing on endurance performance. 

In subgroup analyses, it was found that paracetamol ingestion enhances performance in time-to-exhaustion tests, but such effects were not observed in the analysis focusing on time trials. As mentioned previously, pain reduction is one of the proposed mechanisms by which paracetamol ingestion enhances exercise performance [20]. Therefore, for paracetamol to be ergogenic, it would need to be consumed before exercise that produces high levels of acute muscle pain. Theoretically, due to their open-end structure, it might be that time-to-exhaustion tests produce higher levels of acute pain, which is why paracetamol-induced pain reduction might be ergogenic. However, future work is needed to explore the relationship between pain perception and the effects of paracetamol ingestion in different forms of exercise. Paracetamol ingestion also appears to have effects on the attenuation of neuromuscular fatigue [19]. As recently reported, paracetamol ingestion may increase muscle activation, which might also improve exercise performance [19]. However, such effects were observed in resistance exercise, and it remains unclear if they also contribute to improvements in endurance performance. 

It should be mentioned that the included studies varied in the dose of paracetamol provided to their participants. Specifically, some studies provided paracetamol in doses relative to body mass (e.g., 20 mg/kg of body mass), whereas other used absolute doses from 500 to 1500 mg of paracetamol. The latter dose is likely to be more ergogenic given that paracetamol systemic bioavailability is dose-dependent [29]. Still, the influence of paracetamol dosing needs to be explored in future dose–response studies. Besides paracetamol, more work is also needed on the effects of other analgesic drugs such as tramadol on endurance performance [31,32]. 

The focus of the present review was solely on the ergogenic effects of paracetamol on endurance performance. We did not cover other aspects of paracetamol consumption, such as safety and ethical concerns [20]. Paracetamol is considered to be safe when taken within the recommended doses. However, in some countries, overdosing with paracetamol (doses of 7 g or higher) is one of the most common causes of liver failure [33]. From an exercise perspective, consumption of paracetamol before and after exercise has been reported to attenuate markers of anabolic signaling such as the phosphorylation of ribosomal protein S6, which, depending on the goal of training, also needs to be considered [34]. Ethical concerns are also an issue, as some have voiced their opinion that paracetamol should be included in the World Anti-Doping Agency class of substances subjected to Therapeutic Use Exemption [35]. These important aspects are covered in more detail in a narrative review by Lundberg and Howatson [20].

All included studies used a double-blind study design and were classified as being of good or excellent methodological quality on the PEDro checklist. However, one limitation was observed among the included studies. Specifically, only one of the ten included studies evaluated the effectiveness of the blinding to the placebo and paracetamol trials. In the study that performed this procedure, Tomazini et al. [15] reported that only 14% of the participants correctly identified the paracetamol trial, suggesting that successful blinding occurred. Future studies should consider exploring the effectiveness of the blinding, given that correct supplement identification may impact the outcome of an exercise test and lead to bias in the results [36,37]. While most studies included a familiarization session, two studies [7,13] did not report if a familiarization session was incorporated in the study design. This is important to mention, given that familiarization may impact test reliability [38]. Therefore, future studies should ensure that the participants are adequately familiarized with the exercise test. On a final point, it should be considered that virtually all included studies explored the effects of paracetamol ingestion on endurance performance in males. Therefore, these results should not necessarily be generalized to females, and future studies in this population are needed. Future studies may also consider directly exploring whether sex-specific responses to paracetamol’s effect on endurance performance exist.

## 5. Conclusions

In the present review, we explored the effects of paracetamol ingestion on endurance performance. When we pooled the data among the ten included studies, there was no significant difference between placebo and paracetamol for endurance performance. However, we found an ergogenic effect when we only considered the studies that provided paracetamol 45 to 60 min before exercise. Additionally, paracetamol ingestion enhanced performance in time-to-exhaustion endurance tests, but not in time trials. While paracetamol ingestion may enhance endurance performance, these effects are generally in the range of a trivial to small magnitude. Nevertheless, as this is a fairly novel topic, future research is needed, particularly related to different doses of paracetamol, the timing of ingestion, and various endurance tests. 

## Figures and Tables

**Table 1:** Summary of studies exploring the effects of paracetamol ingestion on endurance performance.

Study	Participants	Paracetamol Dose	Timing of Ingestion before Exercise	Endurance Test	Main Findings
Burtscher et al. 2013	7 male sport science students	500 mg	120 min	Running to exhaustion at 70% of VO_2max_	↔ between conditions
Chagas 2018 ^a^	8 male endurance-trained cyclists	500 mg	60 min	30 min cycling followed by cycling to exhaustion at power output of 80 W, which was increased by 25 W every minute (cadence of 80 rpm)	↔ between conditions
Jessen et al. 2021 ^a^	14 males competing in cycling, triathlon, running, or swimming	1500 mg	60 min	6 min cycling	↔ between conditions
Mauger et al. 2010 ^a^	13 trained male cyclists	1500 mg	60 min	16.1 km cycling time trial	↑ in performance following paracetamol ingestion
Mauger et al. 2014 ^a^	11 recreationally active male participants	20 mg/kg of lean body mass	60 min	Cycling to exhaustion at power output recorded at 70% of VO_2max_	↑ in performance following paracetamol ingestion
Morgan et al. 2019 ^a^	16 active male participants	1000 mg	60 min	3 min all-out cycling	↑ in performance following paracetamol ingestion
Pagotto et al. 2015	12 male runners	20 mg/kg of body mass	45 min	Running to exhaustion at velocity recorded at VO_2max_	↑ in performance following paracetamol ingestion
Pagotto et al. 2018 ^a^	20 male recreationally active runners	1500 mg	45 min	3 km running time trial	↑ in performance following paracetamol ingestion
Tomazini et al. 2020 ^a^	11 male recreational cyclists	20 mg/kg of body mass	60 min	4 km cycling time trial	↔ between conditions
Zandonai et al. 2021 ^a^	29 moderately trained male participants	1500 mg	120 min	40 min constant-work-rate cycling followed by 20 min cycling time trial	↔ between conditions

↑—significant increase; ↔—no significant difference; VO_2max_—maximum rate of oxygen consumption; ^a^—studies included a familiarization session.

## Data Availability

Data used for the meta-analysis are available on request from the corresponding author.
